# Understanding Longitudinal Wood Fiber Ultra-structure for Producing Cellulose Nanofibrils Using Disk Milling with Diluted Acid Prehydrolysis

**DOI:** 10.1038/srep35602

**Published:** 2016-10-31

**Authors:** Yanlin Qin, Xueqing Qiu, J.Y. Zhu

**Affiliations:** 1 School of Chemical Eng. and Light Industry, Guangdong Univ. Technol., Guangzhou, China; 2 School of Chemistry and Chemical Eng., South China Univ. Technology, Guangzhou, China; 3 USDA Forest Service, Forest Products Lab., Madison, WI, USA

## Abstract

Here we used dilute oxalic acid to pretreat a kraft bleached Eucalyptus pulp (BEP) fibers to facilitate mechanical fibrillation in producing cellulose nanofibrils using disk milling with substantial mechanical energy savings. We successfully applied a reaction kinetics based combined hydrolysis factor (*CHF*_*X*_) as a severity factor to quantitatively control xylan dissolution and BEP fibril deploymerization. More importantly, we were able to accurately predict the degree of polymerization (DP) of disk-milled fibrils using *CHF*_*X*_ and milling time or milling energy consumption. Experimentally determined ratio of fibril DP and number mean fibril height (diameter *d*), *DP*/*d*, an aspect ratio measurer, were independent of the processing conditions. Therefore, we hypothesize that cellulose have a longitudinal hierarchical structure as in the lateral direction. Acid hydrolysis and milling did not substantially cut the “natural” chain length of cellulose fibrils. This cellulose longitudinal hierarchical model provides support for using weak acid hydrolysis in the production of cellulose nanofibrils with substantially reduced energy input without negatively affecting fibril mechanical strength.

As one of the most important abundant and renewable biopolymers, cellulose has attracted immense research interest in recent years due to its great potential for producing a variety of high-value products^1^,^2^,^3^. Cellulose nanofibrils (CNF) possess good mechanical properties, low density, thermal stability, and a number of extraordinary properties such as biocompatibility, biodegradability for sustainable utilization in many industries^4^,^5^. CNF can be produced from wood, herbaceous plants, grasses and agricultural residue cell walls. Mechanical fibrillation is the common approach for CNF production as reported in many published studies^6^,^7^,^8^,^9^,^10^,^11^, however, very energy intensive^12^,^13^. A common chemical treatment can reduce energy consumption for producing CNF^14^,^15^, but issues such as chemical recovery need to be addressed for commercial application.

As the main reinforcing constituent in plant cell walls, cellulose is a linear polysaccharide of 1, 4 linked β-D-glucose with a degree of polymerization (DP) of thousands unit and highly hydrogen bonded between inter and intra cellulose chains^16^. Despite current knowledge of cell wall ultra-structure is limited, hemicelluloses, such as xylan, are understood as a matrix component in plant cell wall that glue cellulose fibrils together and coated on cellulose fibril surface^17^,^18^. Furthermore xylan content can be as high as 30% in plant cell walls. Therefore, removing hemicelluloses can play a major role in cell wall deconstruction^19^. To facilitate disintegration of wood fibers into CNF, strong acids were used to remove hemicelluloses^6^,^20^,^21^. These acids also substantially reduced the degree of cellulose polymerization (DP) to result in short fibrils or crystals that negatively affect its performance for polymer reinforcement^11^. Enzymatic treatment is mild and holds promises as green processing, however, most commercial xylanase typically can only remove 30% xylan^22^ and cellulase often greatly reduce cellulose DP^23^. Published work indicate that enzymatic treatments can only produce limited energy savings during subsequent mechanical fibrillation^24^.

Most reported studies on production of CNF through mechanical fibrillation have been qualitative^6^,^7^,^8^,^9^,^10^ due to lack of understanding of cellulose ultrastructure. The information obtained through this type of studies cannot be used to control CNF properties. The objective of this study is to develop a cellulose longitudinal structure model that can be used for achieving quantitative control in CNF production using disk milling with low energy input. Disk milling has the potential for industry scale-up and has been widely used in producing wood fibers commercially. Specifically, we will use a weak acid (oxalic acid) to avoid fibril strength loss by strong acid such as sulfuric acid^11^. We will quantitatively control xylan dissolution and cellulose deploymerization using a combined hydrolysis factor (*CHF*_*X*_) – a reaction kinetics based acid hydrolysis severity^25^, similar to the commonly used combined severity factor (CSF)^26^ but capable of predicting hemicellulose dissolution. Furthermore, fibril DP reduction by hydrolysis as well as by disk-milling will be quantitatively controlled using *CHF*_*X*_ and milling time or energy. A longitudinal wood cellulose structure model will be constructed based on the relationship between measured fibril DP and mean fibril height (or diameter), *DP/d*, so that quantitative prediction of fibril properties such as aspect ratio can be obtained for commercial CNF production.

## Results and Discussion

### Xylan dissolution and cellulose depolymerization

The fraction of xylan retained in water insoluble solids (WIS) after oxalic acid hydrolysis, *X*_*R*_, is determined from the measured xylan content, C_xyls_, and WIS yield, S, as follows,


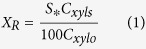


where C_xylo_ = 14.6 is the xylan content of the feed BEP fibers (Table S1). Previously, a combined hydrolysis factor (*CHF*) was developed based on reaction kinetics as a reaction severity. *CHF* is similar to the commonly used combined hydrolysis factor (CSF)^26^, but capable of accurately predicting xylan dissolution when using a bi-phasic xylan model^25^.


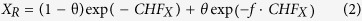



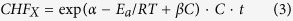


Where *θ* and (*1-θ*) in Eq. 2 were the fractions of the slow and fast reaction (hydrolysis) xylan, respectively. *f* is the ratio of the rate constants between the slow and fast xylan hydrolysis reactions; The subscript X of *CHF* represents xylan. *C* is the initial molar concentration of oxalic acid; *α* and *β* are adjustable parameters, *E*_*a*_ is the apparent activation energy, *R* is the universal gas constant of 8.314 J/mole K, and T is temperature in Kelvin.

Cellulose is also an inhomogeneous material just like xylan that exhibits slow and fast reaction fractions. A bi-phasic model have been used to prediction cellulose dissolution by acid hydrolysis^27^,^28^,^29^. The DP of the hydrolyzed fibers is a good indication of the extent of cellulose deploymerization by an acid, therefore can be fitted by a combined hydrolysis factor *CHF*_*C*_ (subscript C represent cellulose) for cellulose deploymerization, similar to *CHF*_*X*_ for xylan dissolution but with a different activation energy (Fig. S1). However, instead of using two different severities for xylan dissolution and cellulose deploymerization, respectively, we fitted DP using *CHF*_*X*_ according to Eq. (4) to avoid confusion and obtained slow reaction cellulose fractions *θ* ’ and the ratio of reaction rates *f* ’ between slow and fast cellulose fractions.


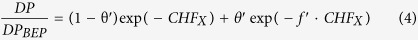


**Figure 1:**
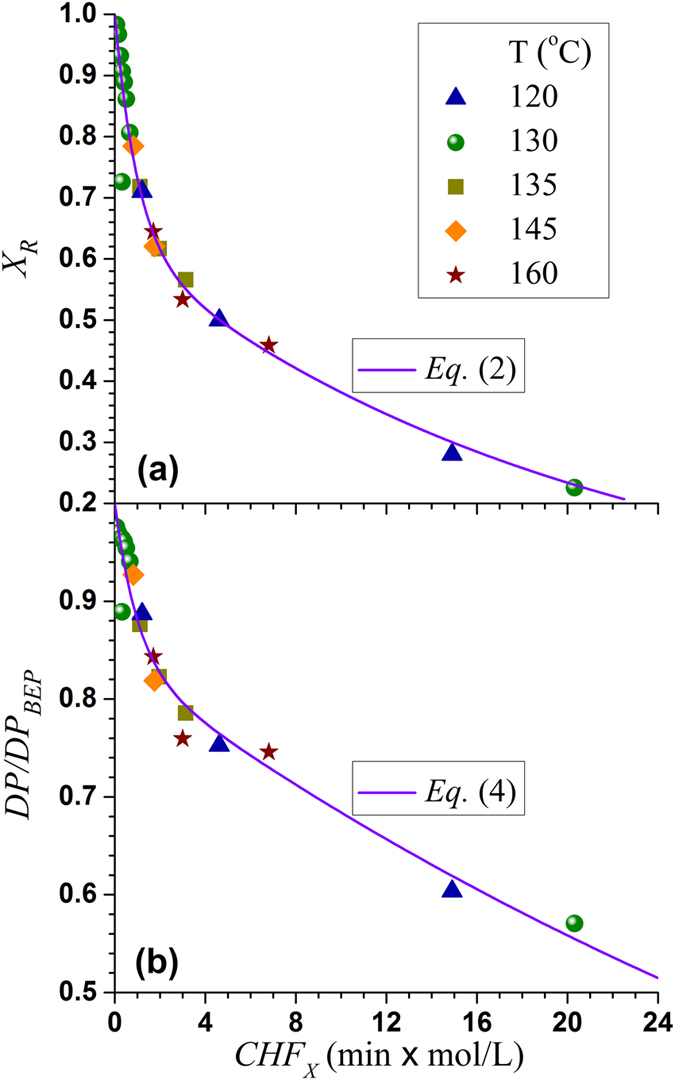
Using the combined hydrolysis factor (*CHF*_*X*_) to predict (**a**) xylan dissolution and (**b**) degree of polymerization (DP) of bleach Eucalyptus pulp fibers by acid hydrolysis in comparisons with experimentally measured fraction xylan retained, *X*_*R*_, and DP.

Excellent fittings of both the xylan dissolution (Fig. 1a) and cellulose fiber depolymerization (Fig. 1b) data were obtained. This indicates that *CHF*_*X*_ is a true reaction severity and can be used to control the degrees of xylan hydrolysis and cellulose deploymeriation rather than using a particular set of reaction conditions for process control. This provides possibility for easing scale-up as demonstrated previously in bioethanol production^30^. The parameters in Eqs (2, 3, 4) were obtained through fitting and listed in Table 1.

### Effect of acid hydrolysis on fibril morphology

**Figure 2:**
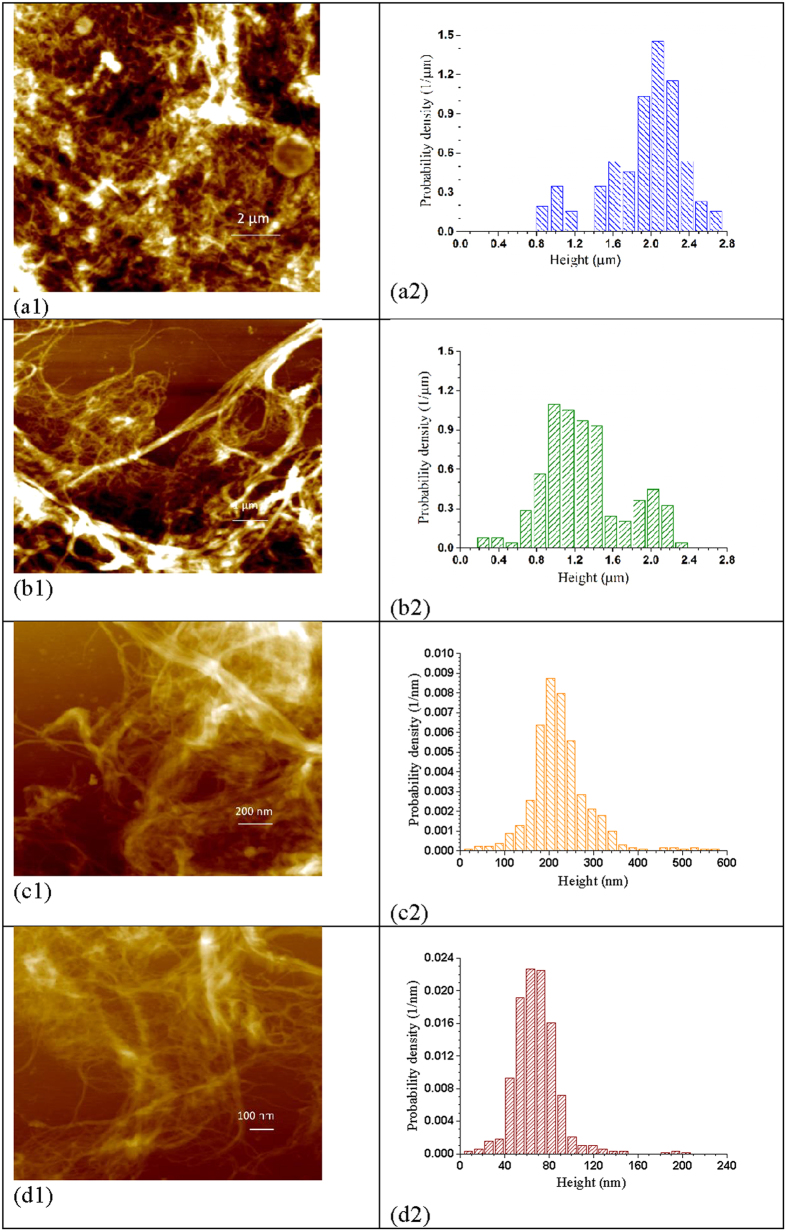
Comparisons of the morphologies (AFM images) and height distributions of cellulose fibrils produced from untreated and acid treated bleach Eucalyptus pulp (BEP) fibers under three severities after 60 min milling in a SuperMassColloider. (**a**) *CHF*_*X*_ = 0 (X_R_ = 1.0), DP = 785 ± 16, scale = 2 μm (untreated BEP); (**b)** *CHF*_*X*_ = 1.21 (X_R_ = 0.72), DP = 638 ± 14, scale = 1 μm; (**c**) *CHF*_*X*_ = 4.62 (X_R_ = 0.50), DP = 485 ± 8, scale = 200 nm; (**d**) *CHF*_*X*_ = 14.90 (X_R_ = 0.31), DP = 286 ± 8, scale = 100 nm.

Three oxalic acid hydrolyzed BEP fiber samples (Nos. 18–20 in Table S1) with varied amounts of xylan content, *X*_*R*_ = 0.72, 0.50 and 0.31, corresponding to *CHF*_*X*_ of 1.21, 4.62, and 14.9, respectively, were used to produce CNF by milling using a SuperMassColloider (SMC). Because acid hydrolysis also depolymerized cellulose, the BEP fiber cellulose DP (before SMC fibrillation) was reduced from 1027 ± 15 to 916 ± 15, 773 ± 14, and 620 ± 15, respectively, for the three hydrolyzed fibers with *X*_*R*_ = 0.72, 0.50 and 0.31. As shown in Table S1, the variation of *CHF*_*X*_ was achieved through the variation of acid concentrations for these three samples. AMF images of the SMC fibrillated (for 1 h) CNF samples, along with AFM measured CNF height (equivalent to diameter) distributions were shown in Fig. 2. Acid hydrolysis facilitated fibrillation. With the increase in hydrolysis severity from *CHF*_*X*_ = 0 (without acid hydrolysis) to 14.90, the fibrils resulted from 1 h SMC milling became finer and more uniform (Fig. 2a1, 2d1). The fibril height distribution quantitatively support the visual observation from the AFM images. The fibrils resulted from untreated BEP fibers (*CHF*_*X*_ = 0) were mainly in micron meter scales peaked at approximately 2 μm (Fig. 2a2). For the hydrolyzed BEP pretreated at *CHF*_*X*_ = 1.21 (*X*_*R*_ = 0.72, DP = 638 ± 14), the SMC-milled fibril heights were reduced but still in the micron meter scales of approximately 1 μm (Fig. 2b2). The fibril heights were substantially reduced to approximately 200 nm (100–350 nm) from the hydrolyzed BEP at *CHF*_*X*_ = 4.62 (*X*_*R*_ = 0.72, DP = 485 ± 8) (Fig. 2c2); furthermore, the distribution was more uniform than the fibrils shown in Fig. 2a2,b2. Further increasing hydrolysis severity to *CHF*_*X*_ = 14.90 (*X*_*R*_ = 0.31, DP = 286 ± 8), fibril heights were further reduced to approximately 70 nm (50–100 nm) with an even more uniform and narrower distribution (Fig. 2d2).

The results in Fig. 2a2d2 indicate that for a given SMC milling of 1 h, uniform fibril height distributions were obtained under the acid hydrolysis conditions which removed 50% or more xylan and depolymerized cellulose to DP 485 or lower. Recall, the BEP has fast xylan content, (1-θ) = 37.7% (Table 1). This suggests complete removal of fast xylan may be necessary to achieve good nanofibrillation to reduce hydrogen bonding^2^. We can also use the model that divides xylan into major (coated) and minor (crosslinking) domains^18^ to visually explain the results in Fig. 2. The minor domain (crosslinking) xylan can be considered the fast reaction xylan that need to be removed to facilitate nanofibrillation. While the major domain xylan coated on cellulose fibril surface are the slow xylan that may pose less barrier to nanofibrillation. In other words, an effective acid (or enzymatic) treatment needs to solubilize the minor domain xylan that crosslinks cellulose fibrils to facilitate nanofibrillation.

**Figure 3:**
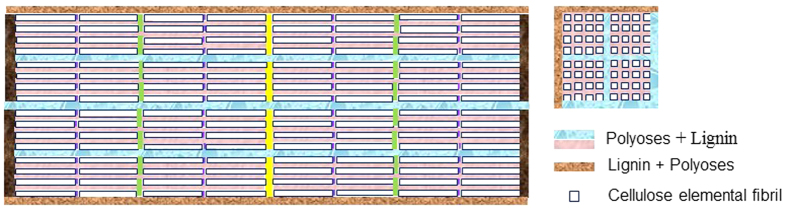
A schematic diagram of the proposed wood fiber longitudinal hierarchical structure model that illustrates the existence of “natural” fibrils with certain length or aspect ratios that vary with fibril lateral dimension (diameter).

We can also use cellulose deploymerization to explain the results shown in Fig. 2a2d2. Wood cell wall has a lateral (radial) hierarchical structure, i.e., wood fibers are made of microfibrils (with DP on the order of 10,000 31) that consists of nanofibrils, and the nanofibrils are made of elemental fibrils (aggregates of multiple cellulose chains based on existing cellulosic cell wall structure models^32^,^33^). We hypothesize that this lateral hierarchical structure model is equally applicable to the fibril length direction. Specifically as schematically shown in Fig. 3, the elemental fibrils in wood are short (i.e., elemental crystal was hypothesized to be 60 nm in early textbooks^34^), but they aggregate to form large and longer nanofibrils on the order of micrometers. The linkage between the two elemental fibrils can be β-1-4, but weak, resemble the so called “more disorder” cellulose^33^, or non β-1-4 glycosidic bonds. We further hypothesize that these nanofibrils further aggregate to form microfibrils on the order of tens of micrometers. The microfibrils form fibers (cell wall tracheid) with length of millimeters. This hypothesis suggests DP reduction is unavoidable and necessary in producing or separating cellulose fibrils at different lateral scales (diameters), as supported by the results in Fig. 2, i.e., feed fibers with lower DP produced finer fibrils. This longitudinal hierarchical model of cell wall tracheid can be corroborated by the fact that endoglucanase treatments which cut fibrils can facilitate cellulose nanofibrils production^7^,^23^,^35^,^36^, just as effective as xylanase treatments that only hydrolyze hemicelluloses and primarily cut lateral crosslinking between fibrils^37^. Moreover, most bleached kraft wood fibers have a DP = 1000–2000, or cellulose chain length of approximately 0.5 – 1 μm (based on the dimension of one glucan unit of 0.5 nm), yet typical length of these fibers are 1000–2000 μm. Therefore, there must exit a longitudinal hierarchical fibril structure to build up these long tracheids in millimeter scale from cellulose fibrils with micrometer scale. Based on this longitudinal tracheid hierarchical model, cellulose deploymerization by acid hydrolysis facilitates the separation of fibrils. This longitudinal hierarchical model needs to be verified in the future through more rigorous studies.

### Spectral transmittance of fibril suspensions

The spectral transmittances of BEP fibril suspensions at 0.1% consistency were measured using a UV-visible spectrophotometer. As shown in Fig. S2, the suspension of the fibril from 1 h SMC milling of the untreated BEP (DP = 1027 ± 15) was opaque with optical transmittance of 0.8% at 600 nm and less than 10% at 1100 nm. Improved fibril separation with the application of acid increased fibril suspension optical transmittance, i.e., fibril suspension transmittances at 600 nm were increased to approximately 5, 12, and 21% at hydrolysis severity *CHF*_*X*_ = 1.21 (*X*_*R*_ = 0.72, DP = 638 ± 14), 4.62 (*X*_*R*_ = 0.50, DP = 485 ± 8), and 14.90 (*X*_*R*_ = 0.31, DP = 286 ± 8), respectively. The transmittances of these three suspensions were 33, 44, and 51% at 1100 nm. These spectral transmittance results are in agreement with the AFM measured fibril size shown in Fig. 2.

### Degree of polymerization and water retention value

**Figure 4:**
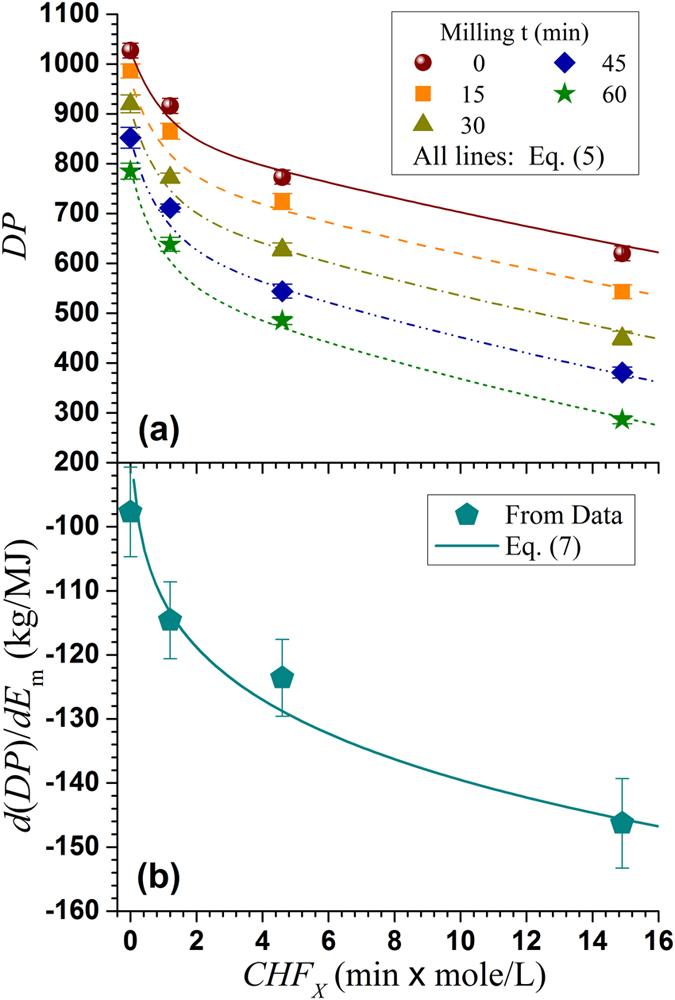
Effects of acid hydrolysis severity *CHF*_*X*_ on fibril DP reduction and mechanical fibrillation energy efficiency through SMC milling. (**a**) DP; (**b**) fibrillation energy efficiency measured by 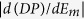.

DP as a measurer of the length and branching of cellulose chains is an important physical-chemical property of cellulose. DP is also an intrinsic parameter of the mechanical properties of cellulose fibrils. Acid hydrolysis can depolymerize cellulose to result in reduced DP in addition to solubilize hemicelluloses as can be seen from the data set of milling time of zero in Fig. 4a. According to the longitudinal hierarchical model of cell wall tracheid presented above, acid catalyzed cellulose deploymerization can greatly facilitate fibril separation for CNF production. Apparently, a severer hydrolysis (greater *CHF*_*X*_) resulted in a cellulose with a greater extent of deploymerization or lower DP. Over hydrolysis can result in fibrils with a substantially lower DP than the “natural” DP of the fibrils and therefore loss of strength, which should be avoided.

DP of the hydrolyzed BEP fibers, however, was still over 600 even at *CHF*_*X*_ = 14.90 with xylan dissolution of approximately 69% (*X*_*R*_ = 0.31). Subsequent mechanical fibrillation by milling also physically broke-up cellulose chains to result in fine and short fibrils with reduced DP^13^. The reduction in fibril DP appears almost linearly with milling time (Fig. 4a) or approximately 60 for every 15 min of milling (equivalent to approximately one pass). Similar reductions in DP of the acid hydrolyzed BEP samples through SMC were also observed, i.e., DP was reduced by approximately 70 for each 15 min of milling. A slightly greater reduction in DP of 80 with 15 min milling was observed for the sample with the greatest xylan dissolution of 69% (*X*_*R*_ = 0.31) at *CHF*_*X*_ = 14.90. Based on these observations, we can express fibril DP using the following equation when combining Eq. (4)


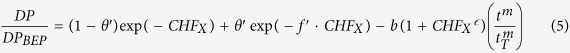


where *t*^*m*^ is SMC milling time and 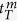 = 60 min is the maximal milling time (a normalizing parameter) of all experiments. Because DP reduction was also linearly proportional to SMC milling energy (Fig. S3), we can express Eq. (5) in terms of milling energy, *E*_m_, as follows:


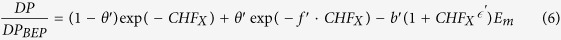


Therefore


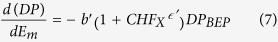


The DP data of the 16 CNF samples produced at 4 different milling times in the study from the 4 feed samples, i.e., the three acid hydrolyzed and the original BEP fibers, along with the DP of these 4 feed samples were used to fit Eqs (5) and (6). The fitting parameters were listed in Table 2. The predicted DP values by Eqs (5) and (6) agree with experimentally measured data very well (Fig. S4). Furthermore, 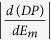, predicted by Eq. (7) were in excellent agreement with experimental data as shown in Fig. 4b.

Equation (6) clearly indicates that the reductions in fibril DP by SMC milling linearly correlated to milling energy consumption, suggesting energy input is perhaps directly related to the break-up of the numbers of glycosidic bond. The absolute values of the slopes of the linear relations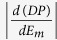, however, were greater for the fibrils produced from acid hydrolyzed fiber samples (Fig. 4b) as indicated by the term 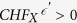 in Eq. (7), and increased with the increase in acid hydrolysis severity *CHF*_*X*_ (Eq. (7) and Fig. 4b). This suggests acid hydrolysis not only depolymerized cellulose or reduced DP (data set with t = 0 in Fig. 4a) but also facilitated subsequent mechanical fibrillation. 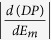 can be used as a measurer of cellulose fibrillation energy efficiency^38^. Equation (7) indicate that acid hydrolysis improved mechanical fibrillation energy efficiency by 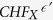 or approximately 160% at hydrolysis severity of *CHF*_*X*_ = 14.9. This is in addition to the DP reduction by acid catalyzed cellulose deploymerization during hydrolysis, i.e., DP was reduced from 1027 to 620 at CHFX = 14.9, clearly indicating the effectiveness of acid pretreatment in facilitating mechanical fibrillation.

WRV is a measurer of cellulose fibril water absorption and swelling ability, or the extent of fibrillation. Fibrillation through disk milling caused delamination and defibrillation of fiber cell wall^13^,^39^ to increase fibril internal surface and external surface area. Acid hydrolysis dissolved hemicelluloses and depolymerized cellulose through the breakage of the intermolecular and the intramolecular hydrogen bonds between hydroxyl groups, which removed a barrier for cellulose swelling^40^ and resulted in increased WRV (Fig. S5). Moreover, the break-up of glycosidic bond by acid catalyzed hydrolysis as well as subsequent mechanical fibrillation allowed easier penetration of the water molecules between the chains to increase WRV, similar to DP (Fig. 1b).

### Fibril aspect ratio

**Figure 5:**
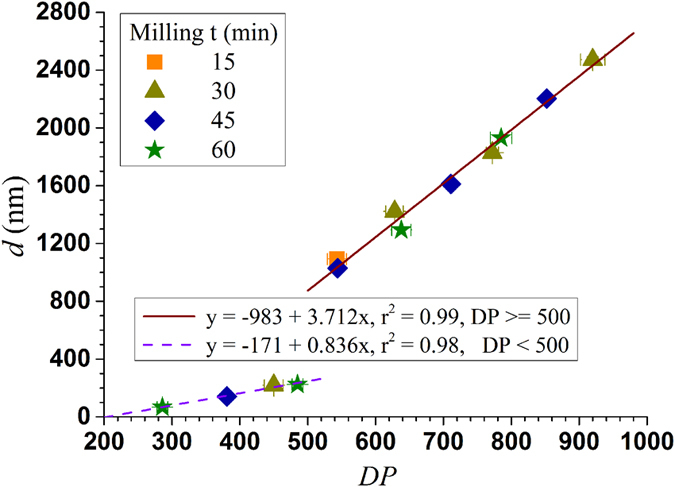
Correlations between fibril DP and AFM measured number mean height (or diameter *d*).

Fibril aspect ratio is a very important morphological parameter especially for polymer reinforcement. Fibril length measurements were difficult using imaging methods for the samples studied here due to entanglement (Fig. 2). DP can be a measurer of fibril length and quantitatively predicted using Eqs (2 and 3). It has long been used as a cellulose chain length measurer related to strength properties of wood fibers in wood fiber science and engineering. Ideally, we would like to separate or produce the “natural” nanofibrils without DP reductions. According to the fiber longitudinal hierarchical model, this “natural” DP varies with the lateral scale (diameter) of the fibril (Fig. 3) as discussed above. AFM imaging can provide statistically meaningful measurements of fibril height that can be treated as diameter. AFM images were taken for all fibrils produced with grinding time over 30 min. The AFM measured number mean heights (diameter) of these fibril samples were plotted against measured DP. Linear relations were obtained as shown in Fig. 5. The correlation for fibrils with DP ≥ 500 has a much greater slope of 3.71 than the correlation for fibrils with DP < 500 of only 0.84. This indicates that before fibril DP reach 500, reduction in fibril diameter was much rapid than reduction in DP through mechanical fibrillation, i.e., a reduction in DP by 100, fibril diameter was reduced by 371 nm, irrespective of the feed fibers were acid hydrolyzed or not. However, once fibril DP reached 500 (corresponding to fibril diameter of approximately 200 nm), diameter reduction by fibrillation becomes difficult, i.e., a reduction in DP by 100 only resulted in a fibril diameter reduction of 84 nm. We believe that this transition exists using any mechanical device or fibrillation method simply due to the amount of energy required for the production of very small particles with very large surface area, as well as the limit of mechanical actions. The specific value of this transition DP or the corresponding fibril diameter of 200 nm may be related to the specific mechanical device used. Further reduction fibril diameter through mechanical means is difficult once a fibril become so small. The fact that the slopes of the correlations between fibril *d* and *DP* shown in Fig. 5 were not affected by acid hydrolysis severity (*CHF*_*X*_) or milling condition, supports the longitudinal hierarchical fiber ultra-structure model schematically shown in Fig. 3. Furthermore, it suggests all BEP fibers were not over-hydrolyzed or milled, i.e., preserved the “natural” fibril DP, even at *CHF*_*X*_ = 14.9 with hydrolyzed fiber DP = 620. This information is very important for using the maximal possible acid hydrolysis severity to maximize energy savings for mechanical fibrillation while preserve fibril strength.

The results shown in Fig. 5 provided a way to quantitatively predict fibril diameter or a fibril aspect ratio measurer (*DP/d*) with the substitution of Eqs (5 and 6) and the correlations in Fig. 5, i.e.


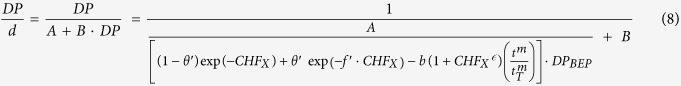



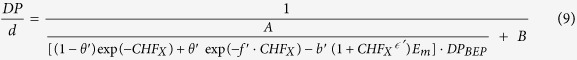


where A and B are listed in Table 2. The utility and validity of this aspect ratio measurer, *DP/d,* need to be verified in terms of its relation to strength properties as well as its versatility to different types of cellulose nanomaterials.

**Figure 6:**
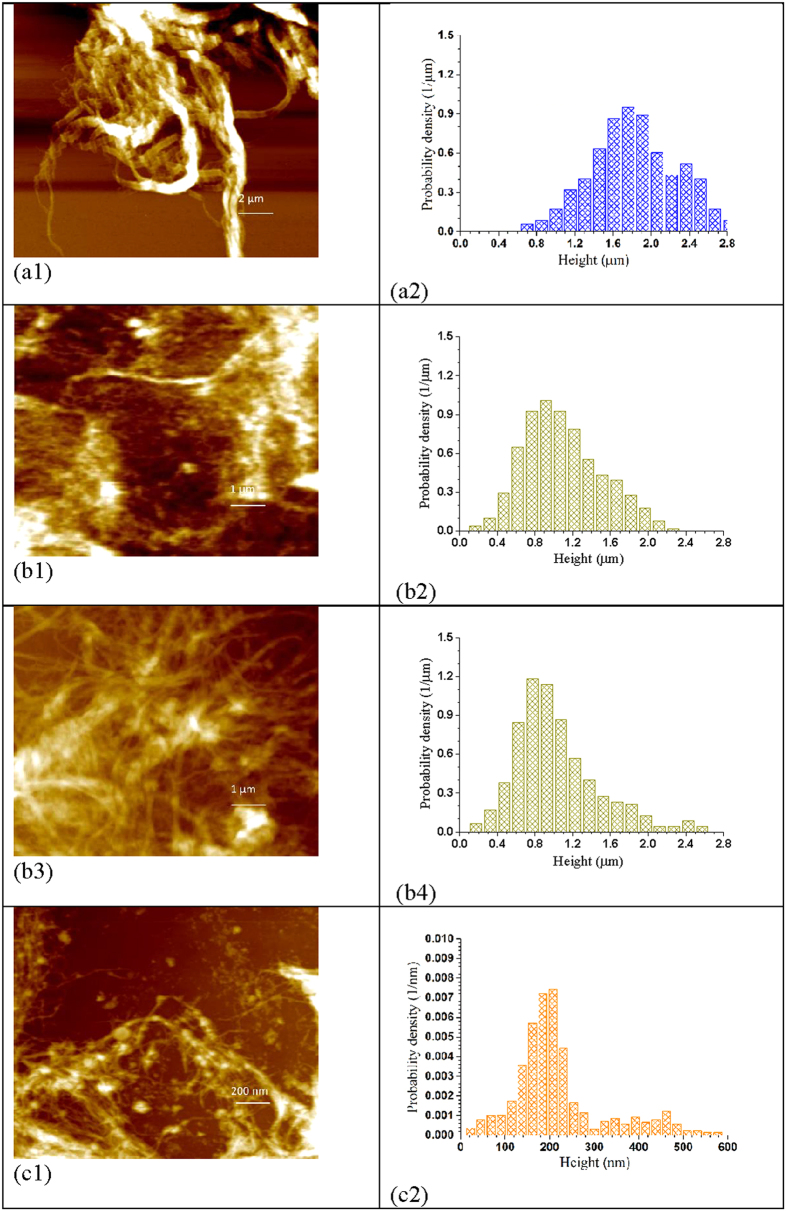
Paired comparisons of the morphologies (AFM images) and height distributions of cellulose fibrils with similar DP but produced under different hydrolysis severities and SMC milling durations. (a1) scale = 2 μm and (a2): DP = 772 ± 9, *CHF*_*X*_ = 1.21 (X_R_ = 0.72), milling t_m_ = 30 min, paired with Fig. 2a1,2a2; (b1) scale = 1 μm and (b2): DP = 543 ± 14, *CHF*_*X*_ = 14.90 (X_R_ = 0.31), milling t_m_ = 15 min, paired with (b3) scale = 1 μm and (b4): DP = 544 ± 14, *CHF*_*X*_ = 4.62 (X_R_ = 0.50), milling t_m_ = 45 min; (c1) scale = 200 nm and (c2): DP = 450 ± 14, *CHF*_*X*_ = 14.90 (X_R_ = 0.31), milling t_m_ = 30 min, paired with Fig. 2c1,2c2.

To support the results shown in Fig. 5, we compared 3 pairs of fibril samples. Each pair had very similar DP but was achieved using different acid hydrolysis severities and SMU milling durations. The selections of the fibrils in each pair are listed in the caption of Fig. 6. In the first pair, the fibril sample shown in Fig. 6a1 was first acid hydrolyzed with 28% xylan dissolution and a DP reduction from 1027 ± 15 (initial BEP fibers) to 916 ± 15, or an acid hydrolysis DP reduction of approximately 110. Mechanical milling further reduced DP to 772 ± 9 or approximately by 140. The second fibril sample was produced without acid hydrolysis and all DP reduction to 785 ± 16 (or approximately 250) was achieved by SMC milling. The fibril height distributions of these two fibrils (Figs 6a2 and 2a2) were similar. The acid hydrolysis produced fibril (Fig. 6a1) having a mean fibril height (diameter) of 1828 nm compared with the purely mechanical fibrillated sample (Fig. 2a1) of mean fibril height 1931 nm. Similar observations were also observed from the other two pairs with samples within each pair were hydrolyzed at different severities, e.g., similar mean diameters, were obtained for the paired fibril samples with similar DP, 1288 nm (Fig. 6b2) vs 1404 nm (Fig. 6b4) and 219 nm (Fig. 6c2) vs 225 nm (Fig. 2c2). These results supports the hypothesis of “natural” fibrils with fixed aspect ratio for a given fibril lateral dimension (Fig. 3). Energy inputs for two fibrils of the same pair with equivalent DP and similar *DP/d*, however, were substantially different. Severer acid hydrolysis resulted in substantial energy savings compared with less severe or no acid hydrolysis, e.g., the energy consumption for producing fibril sample in Fig. 6b1 (DP = 543 ± 14) with acid hydrolysis severity *CHF*_X_ = 14.9 was 0.64 MJ/kg, compared with 1.86 MJ/kg for the fibril sample in Fig. 6b3 (DP = 544 ± 14) with acid hydrolysis severity of *CHF*_X_ = 4.62 (Fig. S3), or a reduction of almost 3 folds. This is simply because longer milling time was used for producing the fibril sample in Fig. 6b3. More energy input was required to mechanically breakdown those glycosidic bonds that otherwise could be hydrolyzed by acid to achieve similar DP as the sample in Fig. 6b1.

## Conclusions

Acid hydrolysis facilitated mechanical fibrillation of wood fibers to produce cellulose nanofibrils with substantially reduced energy consumption using disk milling. Energy savings for mechanical fibrillation was proportional to the amount of hemicellulose dissolution and the extent of cellulose depolymerization. A mathematical expression was developed to accurately predict the degree of polymerization (DP) of disk-milled fibrils as a function of milling time or energy input and the severity of acid hydrolysis represented by a combined hydrolysis factor *CHF*_*X*_. The ratios of fibril DP over mean diameter *DP/d*, as a fibril aspect ratio measurer, obtained from experimental data were linearly correlated to DP irrespective of milling and acid hydrolysis severities. This supports a longitudinal hierarchical cellulose fibril model, i.e., “natural” fibrils exist in wood fibers with certain aspect ratios that vary with the lateral dimensions of the fibrils. Therefore DP reduction is “natural” and unavoidable for producing cellulose nanofibrils. The key to increase fibril aspect ratio is to reduce fibril diameter without cutting the “natural” fibril length. Proper control of acid hydrolysis severity can save mechanical fibrillation energy without substantially reducing fibril aspect ratio to below their natural aspect ratio.

## Materials and Methods

### Materials

Oxalic acid reagent grade were used as received from Sigma-Aldrich (St. Louis, MO). The same bleached kraft eucalyptus dry lab pulp (BEP) from Aracruz Cellulose (Brazil) reported previously^13^,^41^ was used as the CNF feedstock. The chemical composition of the BEP is listed in Table S1. The dry BEP was first soaked in distilled water for 24 h and then disintegrated by a lab disintegrator (Model 73-06-01, TMI, Ronkonkoma, New York) for 20,000 revolutions at 312 rpm and 3% consistency. After vacuum filtration, the pulp with about 30% consistency was further pulped in a mixer using a Hobart mixer (North York, Ontario, Canada). The resultant BEP pulp fibers were collected into a hermetic bag and stored in a freezer for use.

### Acid hydrolysis

BEP fibers were hydrolyzed at 10% solid consistency using oxalic acid at different concentrations and temperatures (Table S1). Hydrolysis experiments were conducted in 1-L reactors and each run used 150 g BEP fibers (in oven dry weight). Three 1-L reactors were placed into a 23-L rotating reactor heated by steam in a jacket in an autoclave configuration as described previously^42^. The digester was rotated at 2 rpm for mixing. At the end of the predetermining duration of reaction, the digester was cooled by flushing tap water into the jacket before opening. The acid hydrolysates were collected for later analyses. The remaining solids were washed several times using distilled water and yields of water insoluble solids (WIS) were determined.

### Analytical methods

A sample of the feed BEP or pretreated WIS was dried and then Wiley-milled to pass a 20 mesh screen and then vacuum dried at 45 °C overnight. The two step sulfuric acid hydrolysis of the Wiley milled solids as described previously^43^ was carried out. Xylose and glucose in the sulfuric hydrolysates were measured using an HPLC equipped with an EconosphereTM C18 column (5-mm particle size, 250 mm × 4.6 mm, Alltech, Deerfield, IL) and a UV1000 ultraviolet detector (277 nm; Thermo Finnigan, San Jose, CA).

### Mechanical nanofibrillation

**Figure 7:**
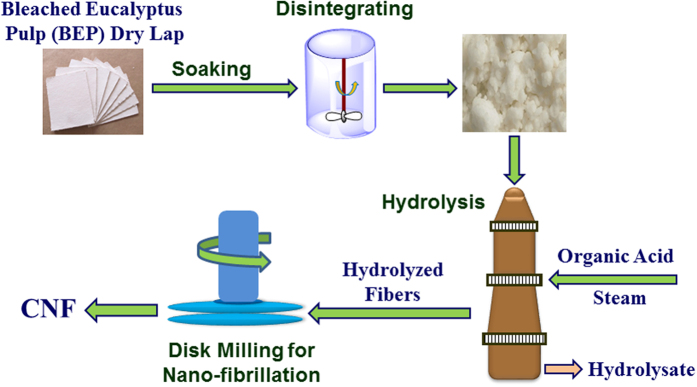
A schematic flow diagram describing the experiments.

Three hydrolyzed fiber samples along with the original BEP fibers of approximately 110 g in oven dry (OD) weight were fibrillated using a stone disk grinder SuperMassColloider (SMC) (Model: MKZA6-2, Disk Model: MKGA6-1632 80#, Masuko Sangyo Co., Ltd, Japan) at solids loading of 2% (w/w) at 1,500 rpm according to previous optimization study^38^. Fiber suspension was fed by gravity continuously through a hopper and pumped using a peristaltic pump (Cole Parmer, Chicago, IL) through a plastic hose. Fibrillation time was 60 min for each run and approximately 200 mL of sample was taken at 15 min interval to obtain time-dependent information. The fibrillated fibril suspension was discharged by centrifugal force, and the time-dependent energy consumption was recorded at 15 min interval. A schematic process flow diagram of pulping, acid hydrolysis, and SMC milling is shown in Fig. 7.

### Atomic force microscope imaging

Atomic force microscope (AFM) images of fibrils were obtained using an AFM (XE-100, Park Systems Corp., Korea) equipped with PPP-NCHR silicon cantilevers in noncontact mode at 25 °C. Fibril specimens were prepared by drying drops of the aqueous fibril slurry of 0.05% g/L on silicon chips. AFM image were zero-order flattened before fibril height determination using a standard algorithm within the AFM system software.

### Optical transmittance

The spectral transparency of fibril suspension was measured using an UV−vis spectrophotometer (Model 8453, Agilent Technologies, Palo Alto, CA) in transmittance mode. Fibril suspension was diluted to 0.1 wt % and thoroughly mixed before placed into a 10 mm quartz cuvette for transmittance measurements.

### Degree of polymerization and water retention value

The degree of polymerization (DP) of the acid hydrolyzed fibers and fibrillated fibrils was measured according to TAPPI Standard Test Method T230 om-08 44. Vacuum dried fibers or fibrils of 0.1 g was added in 10 mL water and then added 10 mL of 1 M cupriethylenediamine solution. The viscosity of the solution was determined using a capillary viscometer with duplicate runs. The DP of the CNF was then calculated according to DP^0.905^ = 0.75(945 log*X*-325), where X is the measured viscosity^45^. The WRV was measured according to a modified Scandinavian test method SCAN-C 62:00 as described previously^46^.

## Additional Information

**How to cite this article**: Qin, Y. *et al*. Understanding Longitudinal Wood Fiber Ultra-structure for Producing Cellulose Nanofibrils Using Disk Milling with Diluted Acid Prehydrolysis. *Sci. Rep.* **6**, 35602; doi: 10.1038/srep35602 (2016).

**Publisher’s note:** Springer Nature remains neutral with regard to jurisdictional claims in published maps and institutional affiliations.

## Supplementary Material

Supplementary Information

## Figures and Tables

**Table 1:** List of xylan dissolution and cellulose deploymerization fitting parameters to Eqs (2) and (4), respectively.

Eq. (2)	Eq. (4)	Unit
*α* = 8.21		None
*β* = 20.7		L/mol
*E*_*a*_ = 25000		J/mol
*θ* = 0.623	*θ’* = 0.838	None
*f* = 0.049	*f’* = 0.020	None

**Table 2:** List of fitting parameters for cellulose deploymerization (DP) fitting parameters to SMC milling time (Eq. (5)) and SMC milling energy consumption (Eq. (6)), respectively, as well for fibril DP and diameter correlations (Eq. (8)).

Eq. (5)	Eq. (6)	Eq. (8) DP ≥ 500	Eq. (8) DP < 500
*b* = 0.1372	*b’* = 0.0543	*A* = −*983*	*A* = −*171*
*ε* = 0.1372	*ε*’ = 0.1766	*B* = *3.71*	*B* = *0.836*
