# Supplementary material for: Understanding Longitudinal Wood Fiber Ultra-structure for Producing Cellulose Nanofibrils Using Disk Milling with Diluted Acid Prehydrolysis
Source: Sci Rep. 2016 Oct 31;6:35602. doi: 10.1038/srep35602 (PMC5086837; doi:10.1038/srep35602)
Supplement: Supplementary Information [file srep35602-s1.doc]

SUPPORTING INFORMATION

**Understanding Longitudinal Wood Fiber Ultra-structure for Producing** **Cellulose Nanofibrils Using Disk Milling with Diluted Acid Prehydrolysis**

Yanlin Qin^1,2^, Xueqing Qiu^1^, J.Y. Zhu^2^*

^1^ School of Chemistry and Chemical Eng., South China Univ. Technology, Guangzhou, China

^2^ USDA Forest Service, Forest Products Lab., Madison, WI, USA

**Total of 7 pages including this cover page**

**Total of one table (Table S1)**

**Total of four figures (Figs. S1-S5)**

Table S1 List of the experimental conditions: oxalic acid concentration (C), temperature (T) and reaction time (t), CHF, along with acid hydrolysis solid yield, xylan and glucan content, and concentrations of xylose and glucose in the hydrolysate.

| **No.** | **C (mol/L)** | **T (K)** | **t (min)** | **CHF_X_** | **Solid yield**  **(%)** | **DP** | **Xylan**  **(%)** | **Glucan**  **(%)** | **Xylose^*^**  **(g/L)** | **Glucose^*^**  **(g/L)** |
| --- | --- | --- | --- | --- | --- | --- | --- | --- | --- | --- |
| BEP |  |  |  |  | 100.00 | 1027 | 14.60±0.26 | 79.40 ±0.87 |  |  |
| 1 | 0.00062 | 403.15 | 60 | 0.08 | 98.24 | 1002 | 14.56 | 80.81 | 0.330 | 0.031 |
| 2 | 0.00123 | 403.15 | 60 | 0.16 | 98.01 | 996 | 14.45 | 80.99 | 0.416 | 0.062 |
| 3 | 0.00185 | 403.15 | 60 | 0.25 | 97.52 | 990 | 13.92 | 81.33 | 1.242 | 0.012 |
| 4 | 0.00247 | 403.15 | 60 | 0.33 | 96.52 | 990 | 13.77 | 82.08 | 1.354 | 0.022 |
| 5 | 0.00309 | 403.15 | 60 | 0.42 | 96.14 | 987 | 13.52 | 82.42 | 2.232 | 0.189 |
| 6 | 0.00370 | 403.15 | 60 | 0.51 | 95.05 | 980 | 13.21 | 83.32 | 2.525 | 0.248 |
| 7 | 0.01235 | 433.15 | 120 | 6.81 | 89.76 | 766 | 7.48 | 87.63 | 9.172 | 0.918 |
| 8 | 0.00617 | 433.15 | 120 | 3.00 | 90.77 | 780 | 8.52 | 86.70 | 8.412 | 0.894 |
| 9 | 0.00370 | 433.15 | 120 | 1.71 | 90.85 | 866 | 10.29 | 86.89 | 6.357 | 0.595 |
| 10 | 0.00370 | 408.15 | 120 | 1.12 | 92.81 | 900 | 11.33 | 85.01 | 4.363 | 0.622 |
| 11 | 0.00617 | 408.15 | 120 | 1.96 | 90.71 | 845 | 9.98 | 86.96 | 6.774 | 0.620 |
| 12 | 0.00926 | 408.15 | 120 | 3.13 | 91.84 | 807 | 9.06 | 85.76 | 7.648 | 0.802 |
| 13 | 0.00123 | 403.15 | 120 | 0.32 | 92.88 | 913 | 11.48 | 85.12 | 4.598 | 0.428 |
| 14 | 0.00247 | 403.15 | 120 | 0.66 | 93.44 | 966 | 12.66 | 84.44 | 3.414 | 0.610 |
| 15 | 0.03704 | 403.15 | 120 | 20.32 | 84.71 | 586 | 3.96 | 92.34 | 13.734 | 1.395 |
| 16 | 0.00309 | 418.15 | 90 | 0.82 | 94.11 | 952 | 12.26 | 83.88 | 3.594 | 0.583 |
| 17 | 0.00617 | 418.15 | 90 | 1.75 | 91.86 | 841 | 9.85 | 85.80 | 7.129 | 0.651 |
| 18 | 0.00942 | 393.15 | 60 | 1.21 | 93.85 | 911 | 11.20 | 84.19 | 4.805 | 0.484 |
| 19 | 0.02572 | 393.15 | 60 | 4.62 | 88.36 | 773 | 8.26 | 89.06 | 8.872 | 0.746 |
| 20 | 0.05012 | 393.15 | 60 | 14.90 | 86.71 | 620 | 5.22 | 90.44 | 12.254 | 0.872 |

* Concentration in the hydrolysate.

Glucan and xylan content in untreated solid fiber were analyzed are 79.42% and 14.60%, respectively.

**Fig. S1.** Fitting of fiber deploymerization by acid hydrolysis to a combined hydrolysis factor
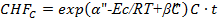
 with *α*” = 10.47, *β*” = 39.2 J/mol and *Ec* = 34300 > E_a_ = 25000 J/mol, in agreement with high xylan dissolution than cellulose in Table S1.

**Fig. S2** Spectral transmittances of the suspensions of cellulose fibrils described in Fig. 3 (SMC milling time = 1 h from BEP fibers pretreated at different severities *CHF*_X_) at 0.1% consistency.

**Fig. S3.** Correlations between fibril DP and grinding energy consumptions of fibrillated fibrils.

**Fig. S4** Comparisons between predicted fibril DP by Eq. (5) and (6), respectively, with those measured.

**Fig. S5.** Time dependent cumulative the WRV of fibrils for the fibrillation.
